# Reply to Herrmann et al. Comment on “van Gemert et al. Asymptomatic Infant Rib Fractures Are Primarily Non-abuse-Related and Should Not Be Used to Assess Physical Child Abuse. *Children* 2023, *10*, 1827”

**DOI:** 10.3390/children11101163

**Published:** 2024-09-25

**Authors:** Martin J. C. van Gemert, Marianne Vlaming, Steven C. Gabaeff, Peter G. J. Nikkels, H. A. Martino Neumann

**Affiliations:** 1 Department of Biomedical Engineering & Physics, Amsterdam University Medical Centers, Location AMC, 1105 AZ Amsterdam, The Netherlands; 2 Private Practice, Criminal Psychology and Law, 6986 CL Angerlo, The Netherlands; marianne@vlamingadvies.com; 3 Clinical Forensic Medicine, Healdsburg, CA 95448, USA; stevengabaeff@gmail.com; 4 Department of Pathology, Wilhelmina Children’s Hospital, University Medical Center, 3584 CX Utrecht, The Netherlands; p.g.j.nikkels@umcutrecht.nl; 5 ZBC-Multicare, 1217 AB Hilversum, The Netherlands; martino.neumann@gmail.com

## 1. General

We thank the authors of [1] for their comments on our paper [2].

Two reasons compelled us to conduct this study. The first reason involved our own experience that pediatricians and pediatric radiologists were falsely certain that, after detecting asymptomatic rib fractures in a child of 3 months, it *must* be child abuse [3]. The second reason related to the bizarre comments of members of the American *Society for Pediatric Radiology Child Abuse Committee* (see our paper [2], reference 8) that it was *grossly irresponsible* and could present a *grave public health risk* that other clinicians reported their findings on the genetic causes of bone fragility and fractures, dismissed as (child abuse) *denialists* ([2], reference 10). For us, it was clear that our study was essential to help resolve this disagreement by addressing our hypothesis that *the incidence of infant rib fractures caused by physical child abuse is significantly lower than the incidence of rib fractures resulting from various other non-abuse causes*.

Although a comment on a published paper can be very stimulating, it is unfortunate that the authors of [1] seem to have missed that our paper [2] is a *hypothesis*, one of the publication categories of this journal, and clearly indicated above the title. The implications are that a full and complete analysis is currently impossible and that approximations combined with assumptions are an obvious requirement and possess evidentiary value. This approach is also scientifically relevant and has, e.g., resulted in the well-known scientific journal *Medical Hypotheses*. Further, it is remarkable that [1] only criticizes our choice of clinical examples rather than addressing the incidence estimates, the core of our analysis.

## 2. Reply to Specific Comments

Chronic placental histiocytic intervillositis correlates with rib fractures because it causes skeletal hypo-ossification, reported in Ref. 43 of [2], which also describes fractures.

The thesis of Güvensel was referred to in the publications of Professor Ulf Högberg [4], a well-respected Swedish scientific author, and we have been requested to provide a copy by several scientific and clinical colleagues. Interestingly, the references used by the clinicians of [3] to (falsely) accuse the child’s parents were mentioned by Güvensel.

The papers regarding which it was unclear to [1] as to why we included them are actually relevant. These included “Amir: on rib fractures in premature infants”; the remaining five focused on infant resuscitation. Thus, modern imaging technologies do not play any role here, and the detection rate of infantile rib fractures had nothing to do with these papers. Thus, the papers contributed perfectly to the questions raised.

Contrary to [1]’s statement, we did make a distinction between symptomatic and asymptomatic rib fractures in the Introduction of [2]: *Physical child abuse related rib fractures will be discussed below in Sections 3.1 and 4, paragraph 4* and in the Discussion, stating the following: *Also, physical child abuse related rib fractures, as well as when caused by resuscitation, are painful since they are traumatic and forceful (personal observations of SCG). Support for this conclusion can be found in Ref. [58] of [3], stating that even infant chest physiotherapy and dressing change (wound care) are painful. In contrast, non-abuse-related cases develop from gradual repetitious deformation in undercalcified ribs from labor contractions, virtually almost always perinatally and, without significant soft tissue damage, are hidden and asymptomatic*.

For the incidence of infant abusive rib fractures in Section 3.1 of [2], we clearly stated that this incidence is unknown in the Netherlands. Also, Dutch child abuse incidences are only determined as a time period average, so this incidence for infants is unknown too. As the “next-best” approach, we therefore used the available Belgian data from the Dutch-speaking part of the southern neighbor country. By using the estimated rib fracture incidence originating from resuscitation procedures in infants, the technique of which can take tens of minutes, we could determine an *upper limit* of the Dutch abuse-related rib fracture incidence, clearly indicated in [2]. This is an appropriate approach for a hypothesis paper and answers our claim, also by scientific standards.

We indeed linked *vitamin D deficiency*, *hypermobile Ehlers–Danlos syndrome*, and *chronic placental histiocytic intervillositis* with osteogenesis imperfecta to deduce an estimated incidence of rib fractures. Here again, the authors of [1] seem to have no concept of what a hypothesis paper is and what it requires to provide reasonable answers.

We stress that the only relevant non-abuse mechanisms with large rib fracture incidences were prematurity and vitamin D insufficiency and deficiency.

Then, let us consider the mentioned meta-analysis by Maguire et al. This paper describes abusive head trauma cases where intracranial injury was present and where abuse had been supposedly confirmed as the cause. This subject is obviously outside the scope of our paper. The statement by [1] that this paper proves wrong that *the identification of such occult rib asymptomatic fractures in infants was frequently an undisputed sign that physical child abuse had taken place* is therefore scientifically unproven.

The so-called *feigned scientific accuracy* of our results is another bold and unproven hypothesis of [1]. Our paper is *not* a piece of opinion but, and we recall, a solid scientific analysis of an important *hypothesis*, already welcomed by many (already read 4112 times as of the 7th of July 2024) and used in court at least 10 times to protect innocent families from unjustified abuse suspicion and conviction. The methodology can be reproduced by other scientists. We thank [1] for suggesting a systematic literature search, and we hope that someone will perform it in the future. Based on our own literature research, we are not afraid of the outcome.

The final paragraph of [1]’s comments begins with unjustified claims. Contrary to [1]’s beliefs, our methodology is scientifically reproducible, substantiates the claim in the title, was understandable for all the authors, and the results are relevant and needed (see our replies above). And, yes, we have hope that rib fractures in infants will no longer be considered an undisputed sign of child abuse. It is remarkable that [1] only states that *this paper is likely to cause harm for the safeguarding of children who have been abused* without considering the significantly more frequently occurring cases of *unjustified* child abuse conviction, which destroy families. The statement that our paper has already caused harm to the journal neglects the many congratulations from clinicians and scientists we have received, and that the result is already being used in courts. We honestly believe that our paper will continue to be referenced frequently, which is what journals hope occurs. The recommendation to consider a retraction is in our opinion just an example of the authors of [1]’s frustration due to their (obvious) incapacity to prove our result wrong, most likely because our result will affect achieving unjustified convictions by child abuse clinicians in court procedures.

In conclusion, the comments in [1] are nevertheless important. First, their wording is essential, *committing a serious offence against scientific diligence and honesty*, because it describes the scientific level of their own comments so well. Second, in our opinion, [1]’s comments strengthen rather than weaken the importance of our paper. It is clear that the authors of [1] cannot tolerate our conclusion because it undermines their way of thinking. Finally, we have already received many congratulations via email, and we have learned that our paper has by now been copied over 4100 times and used at least 10 times in court sessions, one of the reasons for us to undertake the analysis.

## References

[1] Herrmann B., Brüning T., Banaschak S., Berthold O. (2024). Comment on van Gemert et al. Asymptomatic Infant Rib Fractures Are Primarily Non-abuse-Related and Should Not Be Used to Assess Physical Child Abuse. *Children*
**2023**, *10*, 1827. Children.

[2] van Gemert M.J.C., Vlaming M., Gabaeff S.C., Nikkels P.G.J., Neumann H.A.M. (2023). Asymptomatic Infant Rib Fractures Are Primarily Non-abuse-Related and Should Not Be Used to Assess Physical Child Abuse. Children.

[3] van Gemert M.J.C., Zwinderman A.H., van Koppen P.J., Neumann H.A.M., Vlaming M. (2023). Child Abuse, Misdiagnosed by an Expertise Center. Part II. Misuse of Bayes’ Theorem. Children.

[4] Högberg U., Thiblin I. (2021). Rib fractures in infancy, case-series and register case-control study from Sweden. J. Pediatr. Endocrinol. Metab..

